# New Roads Leading to Old Destinations: Efflux Pumps as Targets to Reverse Multidrug Resistance in Bacteria

**DOI:** 10.3390/molecules22030468

**Published:** 2017-03-15

**Authors:** Gabriella Spengler, Annamária Kincses, Márió Gajdács, Leonard Amaral

**Affiliations:** 1 Department of Medical Microbiology and Immunobiology, Faculty of Medicine, University of Szeged, 6720 Szeged, Hungary; kincses.annamaria@med.u-szeged.hu (A.K.); gajdacs.mario@med.u-szeged.hu (M.G.); lamaral@ihmt.unl.pt (L.A.); 2 Travel Medicine, Institute of Hygiene and Tropical Medicine, Universidade Nova de Lisboa, 1349-008 Lisbon, Portugal

**Keywords:** multidrug resistance, multidrug efflux pump, efflux pump inhibitor (EPI), proton motive force, RND pump, ABC-transporter

## Abstract

Multidrug resistance (MDR) has appeared in response to selective pressures resulting from the incorrect use of antibiotics and other antimicrobials. This inappropriate application and mismanagement of antibiotics have led to serious problems in the therapy of infectious diseases. Bacteria can develop resistance by various mechanisms and one of the most important factors resulting in MDR is efflux pump-mediated resistance. Because of the importance of the efflux-related multidrug resistance the development of new therapeutic approaches aiming to inhibit bacterial efflux pumps is a promising way to combat bacteria having over-expressed MDR efflux systems. The definition of an efflux pump inhibitor (EPI) includes the ability to render the bacterium increasingly more sensitive to a given antibiotic or even reverse the multidrug resistant phenotype. In the recent years numerous EPIs have been developed, although so far their clinical application has not yet been achieved due to their in vivo toxicity and side effects. In this review, we aim to give a short overview of efflux mediated resistance in bacteria, EPI compounds of plant and synthetic origin, and the possible methods to investigate and screen EPI compounds in bacterial systems.

## 1. Introduction

The emergence of multidrug resistant bacteria is considered a serious public health threat by the World Health Organization (WHO) and listed as a global risk by the World Economic Forum (WEP) [1,2]. The lack of effective treatment against infections caused by such bacteria requires immediate attention of a cross-sectoral nature, otherwise the mortality rates of people suffering from infectious diseases could soon resemble those as before the discovery of antibiotics [3]. Multidrug resistance (MDR), by definition, is the ability of bacteria to withstand lethal doses of drugs, diverse in their mechanism of action and structure, which would be effective in the elimination of susceptible strains. Efflux pumps (EPs) are proteins, which are constituents of all bacterial plasma membranes, which recognize and extrude antibiotics to the environment prior to reaching their intended targets [4]. Over-expression of EPs is one of the hallmarks for frequent failure of antimicrobial chemotherapy [5]. EP-based resistance in bacteria was first described for the resistance of *Escherichia coli* to tetracyclines via over-expression of Tet proteins [6]. The over-expression of EPs can influence genes encoding the target sites of different antibiotics [7,8,9]. While efflux pumps are ubiquitous in all species of bacteria regardless of the medical importance and pathogenicity of the microorganism, their presence and clinical significance have been studied in detail in a variety of MDR Gram-positive species such as methicillin resistant *Staphylococcus aureus* (MRSA), *Streptococcus pneumoniae*, *Clostridioides difficile*, *Enterococcus* spp., *Listeria monocytogenes*, etc.) and MDR Gram-negative (*Acinetobacter baumannii*, *E. coli*, *Klebsiella pneumoniae*, *Stenotrophomonas maltophilia*, *Campylobacter jejuni*, *Pseudomonas aeruginosa*, *Neisseria gonorrhoeae*, *Vibrio cholerae* and *Salmonella* spp.) bacteria [10,11], as well as in MDR *Mycobacterium tuberculosis* [12].

The antibiotic pipeline is scarce and the shortage of new antimicrobial drugs in development is an increasingly important issue [13,14]. Therefore, investigation of the role of efflux pump systems in drug resistance and the design of efflux pump inhibitors (EPIs) as adjuvant compounds are gaining considerable attention [5,15,16,17,18]. It is important to note that the term EPI should be used as efflux inhibitors (EIs) or efflux modulator (EMs) because various inhibitors or modulators described in the literature do not act on the pump itself.

EP-based mechanisms for MDR have also been demonstrated in MDR fungal infections [19] and MDR cancer [20]. With respect to MDR cancers, the over-expression of EPs has received much attention and the specific efflux pump involved in specific MDR cancer and genes that code for their expression have identified. The presence of such over-expressed proteins such as ABCB1 or P-glycoprotein, ABCC2 or MRP2, ABCG2 or BRCP, etc. has major implications for the clinical outcome for the treatment of many MDR malignancies [21]. The role of efflux proteins in fungal and cancer MDR falls outside of the scope of this paper and the reader is encouraged to visit excellent reviews on these topics [22,23,24,25,26]. In addition, it has been demonstrated that these transporters play an important role in natural ecosystems [27,28].

Although genes coding for efflux pumps may be acquired (example QacA EP carried by plasmids infecting MDR *S. aureus*), the majority of genes coding for efflux pumps in bacteria are found chromosomally and 3%–12% of open reading frames are predicted to encode membrane transport proteins [8,29]. Apart from potential variability from acquisition of plasmid encoded transporters from the environment or horizontal gene transfer (HGT), the same genes can be found in all the species of a given genus, suggesting that they are evolutionarily conserved genes in bacteria [30]. These genes are presented in a highly conserved structure (the genes are frequently organized into operons) and they are subject to tight regulation. Although there is a basal level of expression of these pumps, they are usually in a repressed form without the presence of an effector or activation of the promoter [10]. Efflux pumps most often confer clinically significant drug resistance through over-expression, which may be initially transient as a consequence of sub-inhibitory exposure to a given antibiotic [31]. However, with continued over-expression, in the majority of cases, the presence of insertion sequences upstream of the transporter gene may take place or accumulation of mutations [32] in which several local transcriptional regulators, repressors, or activators as well as global transcriptional regulators can be involved. There are four main regulatory protein families concerned in transcriptional regulation of MDR efflux pumps: AraC, MarR, MerR and TetR (for detailed explanations on the regulation of bacterial efflux pumps, see [33,34]. Nevertheless, antibiotics by themselves are not the principle inducers of the expression of efflux pumps given that bacteria isolated from an environment without recent new antibiotics have demonstrated MDR [35]. In fact, some plant- and soil-associated microorganisms present the highest number of efflux proteins, in an environment where–compared to a clinical setting–the pressure from antimicrobial compounds used in medicine is negligible [36,37]. The fact that there are strains of *P. aeruginosa* with the appropriate pumps to extrude fluoroquinolone antibiotics, isolated before these compounds came to existence provides further evidence for this theory [38].

Six distinct superfamilies of prokaryotic transporters are associated with antibiotic resistance, their structure, the energy source they utilize to transport, and the bacteria in which they occur will be discussed later in this review. Some efflux proteins bestow resistance to just a narrow spectrum of antibiotics (e.g., TetM in *E. coli* against tetracyclines or MexCD-OprJ of *P. aeruginosa* against fourth generation cephalosporins) while others expel out a wide range of antimicrobials (e.g., AdeABC of *A. baumanii*, NorA of *S. aureus* or AcrAB-TolC of *E. coli*) [10]. A plethora of studies are available, demonstrating the promiscuity of these transporters in terms of their substrate specificity [4,15,39]. They have roles in detoxification of endogenous intermediates, metabolic stress responses [28,40,41,42,43,44], removing detergents [45], solvents [46,47], dyes [48], antiseptics [49,50], heavy metals [51,52], bile salts [53], and relevant classes of antibiotics (β-lactams, aminoglycosides, tetracyclines, fluoroquinolones etc.) in the treatment of serious bacterial infections [9]. Some argue that the misuse of biocides (antiseptics and disinfectants) if included in the substrate profile of these pumps, will result in their over-expression and the concomitant cross-resistance to all other pump substrates, including relevant antibiotics [49,54,55]. This review attempts to summarize the relevant literature including the diverse roles that efflux pumps have in bacterial pathogenesis besides the removal of toxic compounds from the cytoplasm.

### 1.1. Efflux Pumps as Relevant Factors in Bacterial Virulence

Numerous reports indicate that the main EP AcrAB-TolC system of *E. coli* (and its corresponding homologs in the related species of *Enterobacteriaceae*) plays a crucial role in the survival and persistence of the microorganism in the gastrointestinal tract, due to its ability to extrude bile, a naturally occurring excretion in the bowel with detergent properties [56,57,58]. Interestingly, the aforementioned efflux pump was one of the first described pumps, in association with the MDR phenotypes of the members of the *Enterobacteriaceae* family. The AcrAB-TolC efflux pump of the enteroaggregative pathotype of *E. coli* is necessary for the bacterium to adhere to HEp-2 cells, also suggesting a role in the pathogenesis of the disease [59]. AcrAB-TolC pump-deleted mutants of *S. enterica* serovar Typhimurium have an impaired ability to colonize poultry or to invade tissue culture cells and macrophages in vitro, and the same pathogen lacking the genes of MacAB efflux pump presented with diminished virulence in an in vivo mouse model [60,61]. The consequence of disrupting the AcrAB-TolC efflux system in the aforementioned bacteria had implications for the expression of the SPI-1 and -2 (*Salmonella* Pathogenicity Islands, each encoding a type III secretion system, having a central role in invasion and systemic infection) as well [62]. Similar results were observed when investigating the virulence of Δ*acrB Enterobacter cloacae* [63], *K. pneumoniae* [64], and Δ*pmrA S. pneumoniae* [65]. Similarly, the CmeABC efflux pump is required for *C. jejuni* to colonize the intestinal tract [53]. Apart from the MexCD-OprJ system, the absence of which caused no impairment to the pathogen, pump mutants of *P. aeruginosa* lacking any other of its efflux systems presented with an attenuated ability to invade Madin–Darby canine kidney (MDCK) epithelial cells. The results of this study were further verified. Once the bacteria were resupplied with the plasmids encoding the pump proteins, their ability to invade the cells was restored [66]. The same impact on the fitness and the effectiveness on colonisation in the genito-urinary tract of female mice were observed by the elimination of the MtrCDE efflux system (by deletion of *mtrD* or *mtrE* genes) of *N. gonorrhoeae* [67,68]. The *acrA*, *acrB* or *tolC*-deficient *Moraxella catarrhalis* strains had reduced invasion levels on nasopharyngeal epithelial cells [69]. In addition, regarding the invasiveness of the pathogen, it has been shown that VexAB, VexCD, VexIJK, and VexGH are required for the expression of genes encoding for toxin-coregulated pilus (TCP) and the cholera toxin (CT) of *V. cholerae* [70]. Interference with the BesABC pump of *Borrelia burgdorferi* had detrimental effects on pathogenicity in mice in a similar way [71]. Also investigated in a murine model, the NorB pump of *S. aureus* seems to play a crucial role in abscess formation [4]. Membrane proteins (proven for MmpL4, MmpL7, MmpL8, and MmpL11; *Mycobacterial membrane protein Large*) of *M. tuberculosis* are associated with increased virulence and survival time in the infected host. Further experiments showed the induction of efflux pump genes inside of macrophages, pointing to a likely role in the intracellular survival of this pathogen [12,72,73,74].

### 1.2. The Role of Efflux Pumps in Biofilm Formation

The prevalence of biofilm formation in infections is estimated to be around 65%, with varying degrees of influence on the course of the disease. Eliminating biofilm-associated bacteria, persisting on catheters, cardiovascular and orthopedic implants present a major problem for clinicians [75]. Biofilm formation–which can also be considered as a passive resistance mechanism–is characterized by modified physicochemical microenvironment by forming a polysaccharide matrix around bacteria, therefore inhibiting the diffusion of antibiotics (making bacteria incomparably more resistant to them) as well as leading to stationary growth and dormancy (attributed to lower oxygen and nutrient levels), making bacteriostatic agents quasi ineffective [76,77,78,79,80]. Several studies described a link between biofilm formation and the activity of efflux pumps suggesting that multiple mechanisms are involved at the same time to contribute to this phenomenon [16,81,82]. Most frequently associated with the involvement in such activities is the Resistance Nodulation and Division (RND) superfamily of bacterial transporters [78]. Defects or inhibition of such pumps with EPIs showed reduction or complete elimination of biofilm formation as well as attenuated virulence in *S. enterica* serovar Typhimurium [83], *E. coli* [84], *S. aureus* [85], *L. monocytogenes* [80], *S. maltophilia* [86], *Burkholderia pseudomallei* [87] and *Klebsiella* spp. [81]. In the same way, investigations centering around biofilm-forming *P. aeruginosa* (a pathogen of particular importance in cystic fibrosis, where the biofilm is a main virulence factor) suggest the role of MexAB-OprM and MexCD-OprJ in the biofilm formation and pronounced resistance in the presence of azithromycin [88], colistin [89], ciprofloxacin [80] and aminoglycosides [90].

### 1.3. Intercommunication between Efflux Pumps and Quorum Sensing

Bacteria use structurally diverse signal molecules (so-called ‘autoinducers’, AI) in cell-cell communications also known as quorum sensing (QS), playing an important role in the expression regulation of certain genes and the ability to adapt to the nature of their surroundings, control the size of the bacterial population in the given environment insuring availability of nutrients [91,92]. It has been hypothesized that besides the structurally diverse group of QS signals such as *N*-acyl homoserine lactones (AHLs) and gamma-butyrolactones (GBLs), antibiotics may also act as QS signaling molecules (QSSMs), having notable effects on the quorum behaviours of bacteria [93]. There is evidence that the QS signaling system and its autoinducer molecules have a regulatory effect on the expression of efflux pump genes. In addition, these pumps export QS signals outside of the bacterial cell, thus they can be considered as one of the determining factors of cell-cell and host-pathogen communication [93,94,95]. Consequently, changes in the expression of MDR efflux pumps can cause alterations in the efficacy of QS signaling. In the case of *P. aeruginosa* [96], over-expression of MexAB-OprM and MexEF-OprN pumps impaired the host infection process, their QS response and resulted in reduced susceptibility to a variety of antibiotics, whereas strains of the same pathogen with defective MexHI-OpmD pumps were incapable of the synthesis of several autoinducers [43]. In contrast, the presence of BpeAB-OprB in *B. pseudomallei* is essential for the synthesis and extrusion of its homoserine lactone-type communication signals, not to mention its role in tissue invasion and cytotoxicity [43]. In *E. coli*, the suppressor of division inhibition (SdiA) protein, which is a QS-dependent regulator of cell division, regulates the expression of the AcrAB pump positively, contributing to the increased minimal inhibitory concentration of several antibiotics [97]. Other studies propose that MDR efflux pumps and QS receptors (e.g., the orphan LuxL homologs) share transcriptional regulators, like in the case of the TetR family of transcriptional regulators [93]. Lastly, the secretion of QS signals is inhibited by EPIs suggesting the importance of the EP system in the QS responses of bacterial population [98].

## 2. Multidrug Transporters

Multidrug transporters are present in almost all bacterial species and they contribute to antibiotic resistance besides their normal physiological functions [99,100]. Bacterial efflux pumps are transporter proteins that are located in the plasma membrane of the bacterium [101]. In bacteria there are six distinct families of transporter proteins: the Small Multidrug Resistance (SMR) family, the Major Facilitator Superfamily (MFS), the Multidrug and Toxic Compound Extrusion (MATE) superfamily, the ATP (adenosine triphosphate)-Binding Cassette (ABC) superfamily and the Resistance Nodulation Division (RND) family [102,103]. Recently, a sixth bacterial efflux pump group was described, namely the Proteobacterial Antimicrobial Compound Efflux (PACE) superfamily [104]. They have protective function in bacteria because efflux pumps remove and extrude the noxious agents from the bacterial cell towards the external environment before they reach their intracellular targets [101]. The substrate specificity of efflux pumps are diverse: they can be specific for one substrate or may export a broad range of structurally different compounds including synthetic and natural antibacterial products [105]. Primary active transporters such as ABC efflux pumps directly use ATP hydrolysis to remove substrates, and secondary active transporters are antiporters, namely SMR, MFS, MATE and RND employ the proton motive force (PMF) and/or sodium motive force as the energy source [106,107].

### 2.1. SMR and PACE Transporters

The SMR secondary multidrug transporters are the smallest known pumps which belong to the drug/metabolite transporter (DMT) superfamily. SMR transporters are typically 100–120 amino acids length and contain four transmembrane (TM) helices with short intrahelical loops and generally function as homodimers [108,109]. These proteins use the proton motive force (H^+^) to extrude noxious compounds (e.g., biocides and disinfectants) out of the bacterial cell [107]. They are found in both Gram-negative and Gram-positive bacterial strains but the most studied model of the family is EmrE isolated from *E. coli* consisting of 110 residues [110]. EmrE operates as an antiparallel homodimer and functionally it is a 12 kDa H^+^/drug antiporter that is capable of transporting many different cations such as ethidium, proflavine, safranin O and methyl viologen as well as erythromycin, and tetracycline [105,111]. Other representatives of the SMR proteins are as follows: EbrAB efflux pump from *Bacillus subtilis*, the QacC and SepA from *S. aureus* [107,112,113]. The newly described PACE multidrug efflux system is the sixth family of bacterial transporters. The model protein AceI (*Acinetobacter* chlorhexidine efflux) from *A. baumannii* is comparable to the size of the SMR family with 150 amino acid long and consists of two tandem transmembrane pair domains [104,105].

### 2.2. MFS Transporters

The MFS is a large family of secondary-active transporters, which are widely distributed in both Gram-positive and Gram-negative bacteria, furthermore they can be found in eukaryotes as well. They use the proton motive force (PMF) as energy source in order to operate in the symport, uniport and antiport of various compounds such as ions, sugars, Krebs-cycle intermediates, phosphate esters, oligosaccharides and antimicrobial agents such as tetracycline and fluoroquinolone [107,108,114,115]. Most members of this family are 400–600 amino acid residues in length and contain 12 or 14 transmembrane α-helical spanners. In Gram-negative bacteria they can form tripartite efflux pump systems with an adaptor and an outer membrane protein, for example EmrAB-TolC from *E. coli* [106,116]. The EmrB transporter protein contains 14 TMS and is attached to the plasma membrane by two fusion proteins. Therefore, the structure of EmrAB involves the transporter protein attached to the plasma membrane by the fusion proteins and it is in turn attached to the TolC protein which offers a conduit for EmrAB extruded substrates [106,114]. EmrA should be present as a hexamer in order to form a continuous channel between EmrB and TolC [117], thus the EmrAB unit shows resistance to hydrophobic uncouplers and antibiotics [114].

In Gram-positive bacteria the MFS family is the most relevant transporter and the best studied MDR pumps are the following: the instrinsic NorA, the plasmid acquired QacA and QacB of *S. aureus*, and LmrP of *Lactococcus lactis* [107]. NorA is a 388-amino-acid protein with 12 transmembrane segments and confers resistance to hydrophilic compounds, biocides, dyes, however it shows only low or no resistance towards hydrophobic drugs [114,118]. A structural and functional homolog of NorA has been described in *B. subtilis* and named Bmr protein. Bmr transporter consists of 12 TM segments and has a similar substrate specificity to that of NorA [119]. QacA and QacB proteins are PMF-dependent *S. aureus* MDR transporters and they have 14 transmembrane segments (TMS) [107,114]. They differ in substrate specificity as QacA can transports to mono- and divalent cations, whereas QacB only exports monovalent substrates [107,120]. The *L. lactis* multidrug transporter LmrP has 12 TMS and these pumps transport lipophilic compounds from the inner leaflet of the membrane to the external environment [114,121]. Other multidrug efflux members of the MFS include PmrA from *S. pneumoniae*, MdeA and SdrM from *S. aureus* [122,123,124].

### 2.3. MATE Transporters

The transporters of the MATE family are the most recently discovered drug efflux pumps, present in both Gram-negative and Gram-positive bacteria [125]. The proteins of this family range from 400 to 700 amino acids in length and present 12-alpha-helical transmembrane regions [108]. MATE transporters are capable to transport structurally dissimilar antibiotics such as norfloxacin, chloramphenicol, ciprofloxacin, kanamycin, ampicillin and many others. In addition, these proteins have the ability to export metformin, cimetidine, ethidium and triethylammonium [125]. Regarding the energy supply of the members in the MATE family two energy sources have been described: the PMF and the sodium ion (Na^+^) gradient [126]. The most well-studied MATE transporter is NorM, first described in *Vibrio parahaemolyticus* (VP), uses a Na^+^-coupled gradient for transporting compounds and contains 12 TMS [127]. There are homologs of NorM in *V. cholerae* (VC), *N. gonorrhoeae* (NG) which are both cation-coupled transporters [128]. NorM efflux pumps can identify a broad range of transport substrates such as dyes, fluoroquinolones and aminoglycosides [129]. H^+^-coupled MATE transporters are found in *Pyrococcus furiosus* (PfMATE) and *Bacillus halodurans* (DinF) [105]. In Gram-positive organisms one of the best-characterised MATE transporter is MepA from *S. aureus*. This is a 12 TMS protein which can recognize hydrophilic fluoroquinolones, biocides, dyes and tigecycline [107]. Additional MATE efflux transporters have been described in the literature such as YdhE from *E. coli*, HmrM from *Haemophilus influenzae*, PmpM from *P. aeruginosa*, CdeA from *C. difficile* and AbeM from *A. baumannii* [107,130].

### 2.4. ABC Transporters

The ATP binding cassette (ABC) transporters implicate a large family of multidrug resistance proteins, found in Gram-negative and -positive bacteria, fungi and eukaryotes. The ABC superfamily is the only multidrug pump family whose members are primary active transporters and utilize ATP hydrolysis for the extrusion of cytotoxic compounds [109]. The ABC transporters are multi-domain proteins because they consist of four units, such as two nucleotide-binding domains (NBDs) and two transmembrane domains (TMDs). The hydrophilic NBDs are the ATPase subunit in the cytoplasm which bind and hydrolyze ATP. The TMDs are highly hydrophobic membrane-embedded domains which usually comprise six transmembrane α-helices and play an important role in recognition and removal of compounds [108,131]. ABC efflux pumps can recognize a wide range of compounds. In Gram-negative bacteria the ABC proteins are organized as tripartite efflux pumps together with a membrane fusion protein and an outer membrane (OM) protein. The most extensively studied bacterial ABC drug exporter is MacB of *E. coli* that operates together with the OM channel TolC and the periplasmic adaptor MacA [106,132].

In Gram-positive bacteria the first bacterial ABC transporter described in the literature was LmrA from *L. lactis* [114,133]. The homodimer transporter LmrA is a 590-amino acid long polypeptide that comprises six transmembrane alpha-helices and a nucleotide-binding domain which is functional as a homodimer [107,134]. Sav1866 from *S. aureus* is a symmetric homodimer protein which served as a model for the study of the operation of the ABC transporter-mediated multidrug efflux. Sav1866 and LmrA are homologous of the human multidrug transporter P-glycoprotein [100,135]. Other ABC family MDR transporters have been discovered such as BmrA protein from *B. subtilis*, EfrAB pump from *Enterococcus faecalis*, PatAB from *S. pneumoniae,* and MsbA from *E. coli* [34,136,137,138].

### 2.5. RND Transporters

The members of the RND family function as proton/drug antiporters, and are the most prevalent efflux transporters localized in the inner membrane of Gram-negative bacteria [100,125,139]. Until recently, scientists believed that RND efflux pumps were only limited to Gram-negative organisms. However, RND pump monomers have been described in Gram-positive bacteria such as *S. aureus*, *Corynebacterium glutamicum*, *C. difficile* and *B. subtilis* [107,140,141].

**Figure 1:**
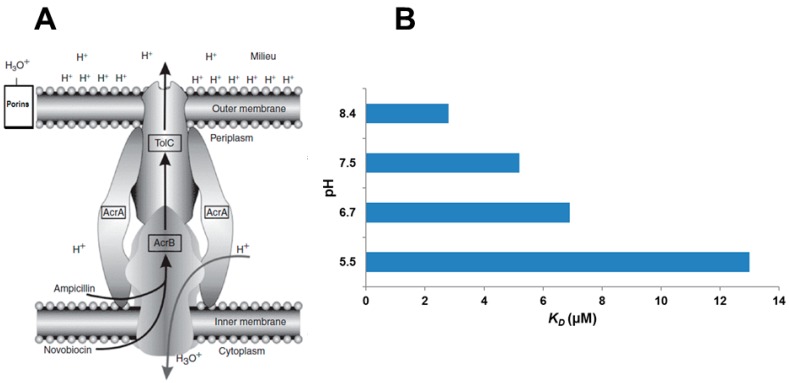
Simplified structure of the AcrAB-TolC pump of a Gram-negative bacterium and proposed mechanism of action ((**A**): adapted from [101], (**B**): adapted from [160]). (**A**) The AcrAB–TolC efflux pump consists of two fusion proteins AcrA (made of six monomers) that anchors the AcrB transporter (made up of three monomers) to the plasma membrane. The latter is connected to the TolC channel (which consist of three monomers, therefore the stoichiometry of the respective pump proteins AcrA–AcrB–TolC is 3:6:3. providing a conduit to the exterior of the bacterium [161]. Noxious agents may enter the transporter from the periplasm or the cytoplasm [128]. The key enzyme is ATP synthase situated within the plasma membrane with one active site for ATP in the periplasm and one active site on the cytoplasmic side of the plasma membrane [162,163]. At pH 7 equilibrium favors the hydrolysis of ATP. At pH 6 equilibrium favors the synthesis of ATP. In order for the pump to work at pH 7, metabolic energy from glycolysis is required [164] for the creation of ATP which is hydrolyzed by the ATP synthase. The product hydronium ion (H_3_O)^+^ present in water enters the AcrAB transporter which had recognized the noxious agent and bound it; this reduces the pH of internal part of the transporter thereby encouraging the dissociation of the agent (**B**) [165] which is then pushed out into the TolC channel by the movement of water and expelled to the milieu. Because the hydronium ions are attracted to components of the outer membrane (lysine, arginine, lipopolysaccharides), they contribute to the proton motive force (PMF) [166]. The AcrAB–TolC pump is now ready to recognize a noxious agent. When the bacterium is in medium of pH 5.5 there is no need for metabolic energy [158]. At this pH the concentration of hydronium ions is high and some are bound to the outer membrane resulting in a strong electrochemical gradient (PMF) that favours their movement into the periplasm via porins [167]. It should be noted that when the AcrAB efflux pump is over-expressed, the major porin C is down-regulated resulting in fewer porins [156]. This provides additional protection from noxious agents.

The most studied RND transporters are the AcrAB-TolC from *E. coli* (Figure 1) and MexAB-OprM from *P. aeruginosa*. In Gram-negative bacteria the RND-type efflux pumps form tripartite complexes which consist of three units such as an inner membrane transporter (AcrB and MexB) that is a secondary active efflux pump of RND family and an outer membrane protein (TolC and OprM) and the periplasmic adaptor protein (AcrA and MexA) connecting the other two transmembrane proteins [105,142,143]. The AcrAB-TolC efflux system forms a large channel spanning the inner membrane, the periplasm and the outer membrane [105,128]. AcrB is an asymmetric homotrimer and has 12 transmembrane α-helices and a large periplasmic subunit, in addition AcrB catalyses drug/H^+^ antiport [108,144,145]. AcrB activity may be controlled by small proteins AcrZ which binds directly to AcrB and regulates the substrate specificity of AcrB [105,106,144,146]. TolC is a homotrimer, functions as a protein channel protein that provides the conduit of compounds extruded to the environment of the bacterium. AcrA is a highly asymmetric hexamer protein which belongs to the membrane fusion protein (MFP) family and is anchored to the inner membrane [130,140,144]. AcrB can mediate extrusion of broad range of substrates. These include cationic dyes such as ethidium bromide, crystal violet, acriflavine, antibiotics such as β-lactams, cephalosporins, fluoroquinolones, macrolides, chloramphenicol, tetracyclines, novobiocin, fusidic acid, oxazolidinones and rifampicin; detergents such as Triton X-100, sodium dodecylsulfate, bile salts and disinfectants [108,130,143,147,148]. The only common feature of these substrates is the lipophilicity or at least the presence of lipophilic domains [143,147,149]. For this reason the AcrB transporter is not able to recognize hydrophilic molecules such as aminoglycosides [139,150] which enter the bacterium via energy dependent transporters. Other RND multidrug transporters have been described in different bacterial species, such as AmrB of *B. pseudomallei*, AcrF (EnvD), AcrD and MdtF (YhiV) of *E. coli*, HI0895 of *H. influenzae*, MexD, MexF, MexY of *P. aeruginosa* or MtrD of *N. gonorrhoeae* [114,130,151,152,153,154].

Based on previous studies RND efflux pumps can be induced to over-expression by two methods:

*Method 1*: Exposure of *E. coli* and *Salmonella* Enteritidis [155,156,157,158,159] to serially increased concentrations of the antibiotic induces the AcrAB-TolC efflux pump to be over-expressed. Such exposure results in the increased activity of genes that code for each component of the RND tripartite AcrAB-TolC [155,156]. The increase of over-expression is accompanied by increases in its resistance to the inducing antibiotic as well as to other classes of antibiotics and detergents [155,156] thereby rendering the bacterium with an MDR phenotype [155,156]. MDR resistance can be completely reversed by phenothiazines which act as indirect inhibitors of the efflux pump supposedly by inhibiting ATP synthase.

*Method 2*: Serial exposure of *E. coli* to a constant sub-MIC concentration of an antibiotic induces the over-expression of the AcrAB-TolC efflux pump [32]. Over-expression continues to take place through as many as 10 serial transfers. Afterwards, whereas continued exposure is accompanied by continued increases of resistance, the activity of the efflux pump has reached its basal level [158]. At this point mutations have accumulated in the 30 S ribosome unit, the plasma membrane and gyrase [32]. This MDR phenotype does not revert and when co-cultured with the wild type counterpart, it cannot compete and within a few serial transfers dies out [32].

Whereas the first method is experimentally useful, it has little relevance to clinical practice since increasing doses of an antibiotic rarely take place. The second method is clinically relevant and serves to explain why increases in resistance as high as 50 times the MIC take place with daily antibiotic therapy at a given dose level. Furthermore, for laboratories that wish to study clinical isolates at the beginning of antibiotic therapy and continuously during prolonged therapy, we suggest that serial MICs should be done for three antibiotic classes to determine the appearance of an MDR phenotype and co-use of small amounts of chlorpromazine to determine the role of efflux pumps in the development of increasing resistance and MDR phenotype. Chlorpromazine inhibits the efflux pump machinery and hence the organism may become susceptible to antibiotics to which it had become resistant during therapy. If chlorpromazine does not reverse resistance, it probably means that the MDR phenotype is due to mutations.

## 3. Methods to Measure the Activity of Bacterial Efflux Pumps

Multidrug efflux pumps are widely distributed and are found in all bacterial species. They are required for bacterial pathogenesis, biofilm formation, furthermore they play an essential role in antibiotic resistance. In order to design and develop efflux pump inhibitors, it is necessary to describe and quantify the activity of efflux pumps under different environmental conditions. The methods for defining efflux pump activity can be divided into two categories: (1) efflux assay, namely the direct measurement of efflux pump substrate transported out of the bacterium; (2) accumulation assay measuring the amount of efflux pump substrate accumulated inside of the bacterial cell [168].

### 3.1. Assays Targeting Efflux Activity

The rationale behind the method that quantifies efflux activity involves preloading the bacterial population with a fluorescent substrate such as ethidium bromide (EB) prior to the efflux assay with and without an efflux pump inhibitor e.g., verapamil or carbonyl cyanide *m*-chlorophenylhydrazone (CCCP). After this loading step the substrate accumulates at maximum concentration within the cell, then the cells are washed in order to remove the substrate and excess inhibitor in the system. After the washing step the energy supply of the cells will be restored, for example by the addition of the energy source such as glucose and the fluorescence will be monitored and recorded within a given period of time on a real-time basis.

There are numerous substrates commonly used in efflux assays. Ethidium bromide is a DNA-intercalating dye that produces fluorescence when it accumulates within the cell. For this reason the intracellular fluorescence is considerably higher than the fluorescence in the extracellular milieu [169,170]. The lipophilic dye 1,2′-dinaphthylamine fluoresces in nonpolar environments and is well retained in membranes [171]. Nile Red is a periplasmic lipophilic dye that binds to phospholipids of the membrane [172]. Regarding the dyes mentioned above 1,2′-dinaphthylamine is the most sensitive, in addition Nile Red and 1,2′-dinaphthylamine are more useful to study RND efflux pumps [168]. Efflux assays can provide information about a molecule whether it is a substrate or not. In addition, the kinetic information of real-time transport and competition for efflux pump binding sites can be readily measured as well [168].

### 3.2. Assays Targeting the Accumulation of EP Substrates

**Figure 2:**
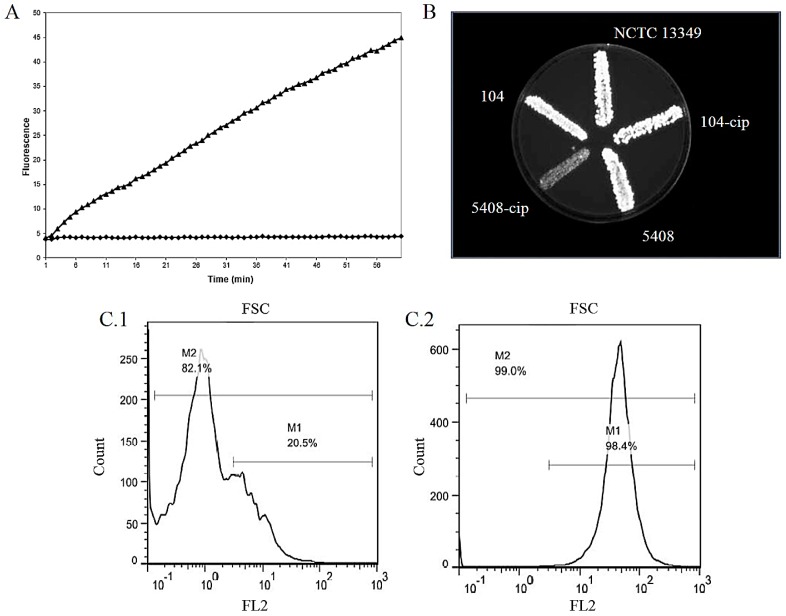
Accumulation methods to measure the activity of bacterial efflux pumps. (**A**) Accumulation of EB by *Salmonella enterica* serovar Enteritidis 104 at pH 5 in the presence of thioridazine (50 µg/mL) using real-time EB method. The flat curve represents the accumulation of the untreated strain [226]; (**B**) Cartwheel method to monitor efflux activity of control (NCTC 13349), clinical (104, 5408), and ciprofloxacin resistant (104-cip, 5408-cip) *Salmonella enterica* serovar Enteritidis strains using EB containing (2.5 mg/L) tryptic soy agar plate [177]; (**C.1**) Accumulation of EB (1 µg/mL) by *E. coli* AG 100 strain using flow cytometry [158]; (**C.2**) Accumulation of EB (1 µg/mL) by *E. coli* AG 100 strain in the presence of thioridazine (20 µg/mL) using flow cytometry [169].

Initially, the accumulation methods were based on the application of radiolabeled substrates but nowadays the assays measure the accumulation of fluorescent dyes. These methods allow the high-throughput screening of efflux pump inhibitors (Figure 2). However, the method is less sensitive compared to the measurement of efflux especially in case of resistant clinical isolates because they have different permeability properties compared to the reference strains [168]. In laboratory practice the fluorescent dyes ethidium bromide (EB) [170] and Hoechst H33342 [173], and naturally fluorescent fluoroquinolones could be used as well. The semi-automated EB assay can monitor the intracellular fluorescence of the dye on a real-time basis [170]. Bisbenzimide H33342 accumulation assay has been described for 96-well plate format using Fluostar Optima (Aylesbury, UK) plate reader [173]. EB accumulation assay for 96-well microplate has been developed to compare the fluorescence of bacteria at different physiological conditions. The fluorescence of the inoculated and incubated EB containing plates are recorded and the plates photographed under UV light in a Gel-doc XR transilluminator (Bio-Rad, Hertfordshire, UK) [156]. Both methods are able to screen numerous samples allowing the comparison of resistant clinical isolates and susceptible reference strains. In order to determine the different bacterial cell subpopulations showing various amount of EB accumulated inside of the cells flow cytometry can be applied. In this case the fluorescence of the cell population will be determined by flow cytometer. EB is excited at 530 nm and the fluorescence is detected through a 585 nm filter (FL-2 channel) [169]. Regarding the efflux pump activity of *P. aeruginosa*, a liquid chromatography/mass spectrometry-based assay was established with ciprofloxacin in order to monitor cold or nonfluorescent compounds [174]. Regarding the rapid development of microfabrication techniques in biology, a fluorescein-di-β-d-galactopyranoside (FDG) based assay has been described using a microfluidic channel device with a fluorescence microscope to study the RND pumps in *E. coli*. Since FDG is hydrolyzed by β-galactosidase in the cytoplasm of *E. coli*, the fluorescent dye, fluorescein will be produced and the signal can be recorded and measured [175]. To allow for rapid and cheap screening, there is a simple, instrument-free, agar-based method that utilizes EB for the demonstration of efflux pump activity in bacteria. The technique can be applied simultaneously to up to twelve bacterial strains to identify clinical isolates that have an over-expressed efflux activity [176,177].

## 4. Efflux Pump Inhibitors

Since efflux mechanisms are essential to remove toxic compounds from bacterial cells and they are important factors in antibiotic resistance, for this reason the inhibition of efflux pump activity is a promising approach overcoming antibiotic resistance [178,179]. Efflux pump inhibitors alone or in combination could restore the sensitivity of resistant strains by increasing the intracellular concentration of antibiotics and decreasing the level of intrinsic resistance. The reason for developing EPIs is the urgent need to discover small molecules, which can be combined with conventional antibiotics in order to block multidrug efflux systems. The first EPI compound MC-207,110 (Phe-Arg-β-napthylamide or PAβN) was described in 2001 that could inhibit the clinically relevant efflux pumps of *P. aeruginosa*, furthermore it proved to be the inhibitor of other RND pumps present in Gram-negative bacteria [180]. Furthermore the authors suggest that MC-207,110 competes with EB for extrusion rather than inhibiting the pump itself. This property of PAβN has been demonstrated by Martins et al. with the use of Michaelis-Menten formula, suggesting that PAβN is not an inhibitor of an efflux pump but rather a competitor of other efflux pump substrates for extrusion [156,181,182,183]. To design effective EPIs, the measurement of kinetic parameters of the inhibitor and substrate and their relationships to the structure of the component of the efflux pump are essential [184]. Thus, one of the most critical issues in seeking for new EPIs is the understanding of how EPIs block the transport of drugs out of the cell [185]. Pagès and Amaral discuss the possible targets of bacterial inhibitors, such as the expression level of genes that induces multidrug resistance (MDR); collapsing energy used by efflux pumps; inhibiting their functional assembly or the blockage of the outer membrane channel [186]. Molecular simulations can provide information about the ligand-binding process in efflux pumps (e.g., AcrB) and possible action mechanism of inhibitors [187]. Furthermore, EPIs should have little or no antibacterial activity when applied alone, but in combination with antibiotics, they should have a synergistic effect. Concerning the chemical nature of EPIs, they are compounds from natural (plant) sources, semi-synthetic derivatives of existing EPIs, and fully synthetic EPIs as well.

### 4.1. EPIs of Plant Origin

**Figure 3:**
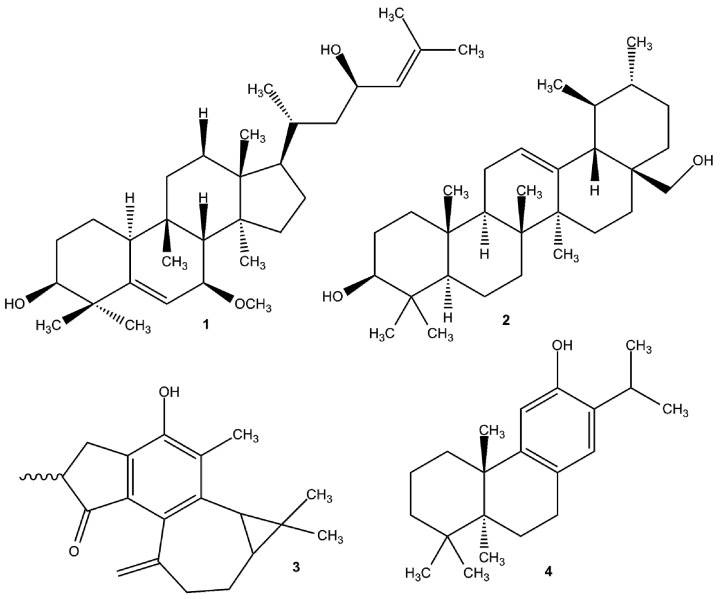
Examples for natural EPI compounds. (**1**) Karavilagenin C; (**2**) uvaol; (**3**) jatropholone A and B; (**4**) ferruginol.

Recently, the use of plant-derived EPIs such as tetrandrine and liquiritin against fluoroquinolone transporters in *E. coli* has been patented. In addition, a geraniol EPI has been developed as well [188]. Regarding the literature, there are excellent reviews describing the most important bacterial efflux pump inhibitors derived from plant sources (Figure 3) [188,189,190]. The present review gives some important examples concerning EPI compounds in Gram-positive and Gram-negative bacteria.

Berberine was isolated from *Berberis fremontii* and since plants can produce antimicrobial agents in order to protect themselves from pathogens, isolating natural EPI from *Berberis* species seemed to be a possible solution. It has been shown by the group of Lewis et al. that *Berberis fremontii* used in Native American traditional medicine produces a potent EPI 5′-methoxyhydnocarpin (5′-MHC) that could inhibit the NorA activity in *S. aureus* restoring its sensitivity against quinolones [191]. However, due to its toxicity, it has no clinical future.

*Trans,trans*-1,7-diphenylhepta-4,6-dien-3-one isolated from *Alpinia katsumadai*, (*Zingiberaceae*) has weak antimycobacterial activity and strong efflux pump inhibitory effect in *Mycobacterium smegmatis* [192]. Sarothrin (5,7,4′-trihydroxy-3,6,8-trimethoxyflavone) isolated from *Alkanna orientalis* (L.) Boiss. (*Boraginaceae*) leaf and flower extract exerted efflux pump inhibitory activity against NorA of *S. aureus* [193]. Capsaicin from *Capsicum annuum* L. (*Solanaceae*) inhibits the NorA efflux pump of *S. aureus* and has the ability to reduce the virulence of *S. aureus* inhibiting the invasiveness [194].

*Carpobrotus edulis* (L.) N.E.Br. (*Aizoaceae*) is a great source of EPI compounds. The compound uvaol isolated from *C. edulis* reduces resistance of the MRSA COL_OXA_ strain to oxacillin, the antibiotic to which it was initially resistant. Furthermore it could inhibit the efflux pump system of this organism. Oleanolic acid also isolated from *C. edulis* was active in modulating accumulation and efflux of EB in *E. coli* strain [195]. The members of *Euphorbiaceae* family represent a valuable source of antimicrobial and efflux pump inhibiting agents in bacteria and cancer cells. A penta-substituted pyridine derivative 2,6-dimethyl-4-phenylpyridine-3,5-dicarboxylic acid diethyl ester from *Jatropha elliptica* (*Euphorbiaceae*) without any antibacterial effect improves the activity of ciprofloxacin and norfloxacin against *S. aureus* [196]. The terpenoids of *Euphorbia hirta* can contribute to membrane destruction and biofilm cell detachment in *P. aeruginosa* [197].

Inhibition of NorA can be carried out using olympicin A isolated from *Hypericum olympicum* L. cf. uniflorum [198] or reserpine isolated from *Rauwolfia vomitoria* [199], furthermore carnosic acid from *Rosmarinus officinalis* [200] and ferruginol from *Chamaecyparis lawsoniana* can block NorA-induced EB efflux from NorA over-expressing *S. aureus* [201]. Coumarins isolated from the tropical tree *Mesua ferrea* (*Guttiferae*) can inhibit the NorA efflux pump of *S. aureus* [202].

*Momordica balsamina* L. (*Cucurbitaceae*) is an African medicinal plant with bioactive EPI compounds, such as karavilagenin C with efflux inhibiting activity on MRSA COL_OXA_ and balsaminagenin B against efflux systems of *E. faecalis* ATCC 29212 [203].

It can be concluded that natural compounds are more effective on Gram-positive bacteria due to the membrane transporters of the MFS, SMR or ABC superfamilies. In contrary, the transporters present in Gram-negatives are more complex because of the permeability barrier of the outer membrane. For further clinical applications EPIs should target only bacterial efflux pumps since there are compounds that can inhibit human transporters as well [204].

### 4.2. EPIs of Synthetic Origin

**Figure 4:**
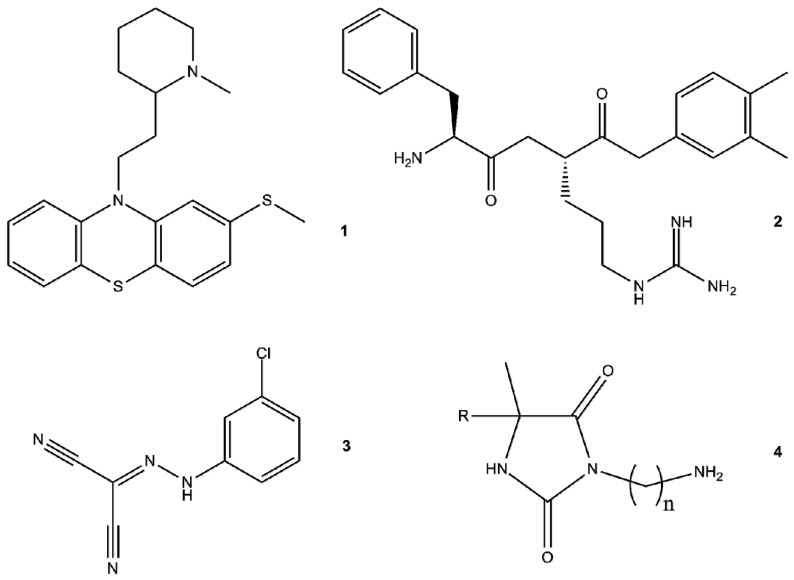
Examples of synthetic EPI compounds. (**1**) Thioridazine; (**2**) Phenylalanine-arginine β-naphthylamide (PAβN); (**3**) carbonyl cyanide 3-chlorophenylhydrazone (CCCP); (**4**) general structure of EPI hydantoins.

The use of efflux pumps inhibitors (EPIs) in order to improve the activity of antibiotics has been investigated [205]. Besides antibiotics there are numerous molecules with antibacterial effects. These compounds are termed “non-antibiotics” and they can potentiate the activity of antibiotics by altering the permeability of bacteria as “helper compounds” or they can exert immunomodulating effect enhancing the killing activity of macrophages as “macrophage modulators” (Figure 4) [206].

Phenothiazines represent one of the most important class of EPI non-antibiotics. Since phenothiazines can act as electron donors on the cytoplasmic side of the plasma membrane, hyperpolarization results and membrane-linked processes are inhibited and phenothiazines can interfere with numerous cellular processes. Promethazine (PMZ) showed synergistic effect with gentamycin due to its antiplasmid effect to cure recurrent pyelonephritis caused by resistant *E. coli* [207]. Thioridazine (TZ) eliminates intracellular *M. tuberculosis* and TZ can be considered for MDR-TB or extensively drug-resistant XDR-TB [208,209]. Chlorpromazine (CPZ) is a well-known EPI compound and its activity is due to its indirect effects on ATPase activity that is dependent upon Ca^2+^ [210]. Newly developed *N*-hydroxyalkyl-2-aminophenothiazines are effective EPIs of the AcrAB-TolC system of *E. coli* K-12 AG100 [211].

Hydantoin derivatives possess a wide range of biochemical effects as well as various pharmacological properties, e.g., efflux pump inhibiting activity in Gram-positive bacteria [212] and arylhydantoins can inhibit the AcrB pump of *E. aerogenes* [212]. It has been shown that some hydantoin derivatives are valuable inhibitors of the ABCB1 transporter of mouse T-lymphoma cells transfected with the human *ABCB1* gene. In addition, some of these hydantoin derivatives reverse or reduce resistance mouse lymphoma cell line to cytotoxic agents to which they are initially resistant [213].

The organosilicon compound SILA-421 (1,3-dimethyl-1,3-bis(4-fluorophenyl)-1,3-bis{3-[1(4-butylpiperazinyl)]-propyl}-disiloxane tetrahydrochloride) was developed as an anticancer agent modulating the ABCB1 transporter of cancer cells [214]. Subsequently, its role as adjunct for the therapy of antibiotic-resistant *E. coli* infections conferring plasmid mediated resistance has been demonstrated [215]. Martins et al. proved that SILA 412 enhances the killing of intracellular extensively drug-resistant tuberculosis (XDR-TB) [216].

The pyranopyridine compound MBX2319 and its more potent derivatives act through competitive inhibition and/or blockage of access to the substrate binding site of the RND class AcrAB-TolC efflux pump in *E. coli* and other pathogens of the *Enterobacteriaceae* [217,218].

Several compounds, such as globomycin (inhibitor of lipoprotein-precursor-processing enzyme), carbonyl cyanide *m*-chlorophenyl hydrazone or CCCP (energy uncoupler, inhibits the proton motive force), quinolines and arylpiperazine derivatives have been identified as compounds to reverse MDR in *E. coli* over-expressing efflux pumps [218]. Among the arylpiperazines, the naphthyl derivative 1-(1-naphthylmethyl)piperazine, could enhance the susceptibility of MDR *E. coli* to fluoroquinolones and increased the intracellular concentration of levofloxacin and EB [220].

In addition, analogues of antibiotics have been patented as efflux pump inhibitors such as tetracycline analogues, analogues of the aminoglycoside paromomycin, and fluoroquinolone analogues can be applied to inhibit efflux pumps [205].

Trifluoromethyl ketones have been shown to interfere with numerous virulence factors of bacteria due the the inhibition of the PMF: 1-(2-benzoxazolyl)-3,3,3-trifluoro-2-propanone inhibits of motility in *Helicobacter pylori* [221], other derivatives inhibit the QS response and the efflux related resistance in *E. coli* [222]. Fluorinated β-diketophosphorus ylides have been tested for EPI activity against the AcrAB-TolC system of *E. coli* and it has been demonstrated that the most potent derivatives reduced the expression of efflux pump and quorum sensing (QS) genes [223].

## 5. Concluding Remarks

Multidrug efflux pumps have great importance regarding multidrug resistance in bacteria and they are involved in various cellular processes such as biofilm formation and QS. The expression of efflux pumps depends on growth rate, population density, starvation, accumulation of metabolites, and pH of the milieu. The majority of the EPIs can interfere with biofilm production, e.g., TZ, NMP, and PAβN can reduce or abolish biofilm formation [224]. Unfortunately, to date, no active EPI has been introduced into the clinical practice due to the low selectivity, low stability, high cytotoxicity and the strong pharmacological effects of these inhibitors in eukaryotic systems, especially in the human host [225].
